# The signature genes of cuproptosis associates with tumor immune microenvironment and predicts prognosis in kidney renal clear cell carcinoma

**DOI:** 10.3389/fonc.2024.1409620

**Published:** 2024-08-14

**Authors:** Shuhan Liu, Shijie Lv, Xi Li, Weiguo Lu, Shengjie Chen

**Affiliations:** ^1^ Department of Clinical Laboratory, The First Affiliated Hospital of Guangzhou University of Chinese Medicine, Guangzhou, Guangdong, China; ^2^ Department of Pharmacology, School of Medicine, Jinan University, Guangzhou, Guangdong, China

**Keywords:** cuproptosis, CRGs, tumor immune microenvironment, Fdx1, KIRC

## Abstract

**Background:**

Cuproptosis is a new form of cell death, which has great potential to be developed in tumors treatment. Our study aimed to explore the predictive value of cuproptosis-related genes (CRGs) in various cancers, with a focus on kidney renal clear cell carcinoma (KIRC).

**Method:**

A total of 9502 pan-cancer patients from TCGA cohort were enrolled. The relationships between CRGs and overall survival (OS) or disease-free survival (DFS) were analyzed. Gene Set Variation Analysis (GSVA) enrichment analysis was performed to explore the expression differences of CRGs. Multivariate Cox regression analysis was used to evaluate the association between GSVA scores and patient survival. KEGG and GO analyses were employed to identify the biological functions and pathways. The expression and prognostic characteristics of FDX1 were examined to evaluate the correlation between FDX1 and KIRC. Cell experiments were conducted to verify whether FDX1 was involved in cuproptosis of Caki-1 cells induced by Elesclomol.

**Results:**

Positive cuproptosis signature genes(pos.cu.sig) exhibited the correlation with prognosis in KIRC, and all of these genes showed differential expression between KIRC and normal tissues. The GSVA score of pos.cu.sig was associated with excellent survival (HR=0.61, *P*<0.05), which can also serve as an independent prognostic factor for KIRC. There was a close correlation between pos.cu.sig and the tumor immune microenvironment in KIRC by KEGG and GO analysis. FDX1 expression was correlated with KIRC grade and positively associated with prognosis in KIRC patients. Compared with the control group, cell proliferation and migration were significantly inhibited, FDX1 expression was up-regulated, and Fe-S cluster protein content was decreased of Caki-1 cells after Elesclomol treatment.

**Conclusions:**

This study provides compelling evidence that cuproptosis is closely linked to the prognosis of KIRC. FDX1 holds promise as a viable biomarker and therapeutic target for assessing the effectiveness of tumor immunotherapy in KIRC.

## 1 Introduction

Copper is an essential trace element involved in various biological processes. Cuproptosis, a copper-dependent form of regulated cell death, is regulated by mitochondrial ferredoxin 1-mediated protein lipoylation which is different from autophagic cell death or traditional apoptosis or necrosis (1). Recent studies showed that the copper levels of both serum and tumor tissues were significantly increased in cancer patients compared to healthy counterparts (2). Maria et al (3) further proved that dysregulation of copper homeostasis may trigger cytotoxicity, alterations in intracellular copper levels may influence the development and progression of cancer.

Cuproptosis regulatory genes are closely related to tumor occurrence and progression (4). Previous studies have reported that cuproptosis plays a vital role in cancer, they screen out a series of cuproptosis-related genes (CRGs) through whole-genome CRISPR-Cas9 selection screen, some CRGs conferred resistance to cuproptosis, such as FDX1, LIPT1, LIAS, DLD, PDHA1, DLAT and PDHB;other CRGs sensitized the cells to cuproptosis, including CDKN2A, GLS and MTF1 (1). However, whether these CRGs are correlated with cancer patient prognosis remains largely unknown. Several genes involved in cuproptosis were identified, which may offer novel strategies to predict the prognosis of cancer patients.

A pan-cancer mRNA expression profile and clinical data were downloaded from The Cancer Genome Atlas (TCGA) database in the present study. CRGs were then used to construct a prognostic multigene signature for cancer patients’ survival. Overall, our data indicate that CRGs are correlated with prognosis of KIRC and tumor immune microenvironment, and may be prognostic markers and potential therapeutic targets for KIRC.

## 2 Materials and methods

### 2.1 Data collection

A total of 9502 pan-cancer patient samples with publicly available RNA sequencing data were downloaded from the TCGA website (https://gdc.cancer.gov/). The gene expression profiles in the TCGA dataset were identified using the “limma” R package. Data from TCGA are publicly available, so no ethics committee approval was required for this study (5).

### 2.2 CRGs acquisition

Ten CRGs were acquired from previous research (1).

### 2.3 Gene set variation analysis

“GSVA” R packages were used to perform GSVA enrichment analysis in heatmaps. The GSVA analysis was conducted using the “c2.cp.kegg.v6.2.symbols” file downloaded from the MSigDB database.

### 2.4 Gene set enrichment analysis

To investigate the functional enrichment of the prognostic signature, we used GSEA (http://www.broadinstitute.org/gsea/index.jsp). We considered pathways related to immune and progression of KIRC to be significantly enriched when a P-value was less than 0.05 and the false discovery rate was less than 0.05 (6).

### 2.5 Functional enrichment analysis

A Kyoto Encyclopedia of Genes and Genomes (KEGG) and Gene Ontology (GO) analysis based on the DEGs (|log2FC|≥1, FDR < 0.05) between high- and low-score groups was conducted using the “clusterProfiler” R package. With the R package “gsva”, the infiltrating score of 19 immune cells and 22 immune-related functions was calculated using single-sample gene set enrichment analysis (ssGSEA).

### 2.6 Validation of the prognostic signature

According to the median GSVA score of the pos.cu.sig. gene, KIRC patients were divided into subgroups with low and high scores. The survival analysis was conducted using Kaplan-Meier survival analysis using the R package survminer. The prognostic factors for OS of patients with KIRC were assessed using univariate and multivariate Cox analyses. In addition, a nomogram was developed to further evaluate this prognostic signature’s predictive power (6).

### 2.7 Cell culture

KIRC cells (Caki-1) were obtained from the Cell Bank of Chinese Academy of Sciences (Shanghai, China). Caki-1 cells were cultured in McCoy’s 5A medium (Gibco, #12330031) supplemented with 10% fetal bovine serum (Cegrogen, #21014040).

### 2.8 Cell counting kit-8 assay

Caki-1 cells were seeded into 96-well plates and exposed to Elesclomol (MCE, # HY-12040) for the indicated hours. Then, 10 μL of CCK-8(ABKBio, #ABK0011C) was added to the cells, and the cells were cultured at 37°C for 4 hours. Then, the absorbance (OD value) at wavelengths of 450nm was measured with a microplate reader (Thermo, #1410101).

### 2.9 EdU proliferation assay (imaging and cytometry)

Detection of Elesclomol effect on Caki-1 cells proliferation was performed according to the manual of Hyper Fluor™ 488 azide EdU Imaging Kits (APExBIO, #K2240) and 6-FAM Azide EdU Flow Cytometry Assay Kits (APExBIO, #K1176). After treatment by Elesclomol, 10 μM EdU labeling medium was added to the cell culture to finish co-incubation for two hours. Next, cells were washed with PBS and fixed with 4% paraformaldehyde for 15 min. After washed with PBS and incubated by 0.5% TritonX-100 for 10 min, anti-EdU working solution was added to stain at room temperature for 30 min. Subsequently, the DNA contents were stained with 5 μg/ml Hoechst 33342 for 30 min. The stained cells were observed under a fluorescent microscope (Leica Microsystems) or resuspended and analyzed on the flow cytometer (BD Bioscience), and data were processed using FlowJo and calculated the average fluorescence intensity.

### 2.10 Scratch assay

Cells were cultured in a 6 well plate to a confluent monolayer. The cell monolayer was scratched in a straight line with a p200 pipette tip. The culture medium was removed and washed (two times) and replaced with 2 mL culture medium. Images were taken at initial scratch and 24、48 hours later.

### 2.11 Quantitative real-time polymerase chain reaction assay

Total RNA was extracted using ABKzol (ABKBio, #ABK0005M). cDNA was synthesized from 1μg total RNA using the ABKscript RT MasterMix (OneStep gDNA Removal) (ABKBio, #ABK0004M), and the ABKuniversal SYBR Green qPCR Mix (ABKBio, #ABK0003M) was used for quantitative reverse transcription polymerase chain reaction (qRT-PCR). For analysis, the threshold cycle (Ct) values for each gene were normalized to expression levels of β-Actin. All primers were designed and synthesized by Shangya Biotechnology. The primer sequences used in PCR are as follows: FDX1:5’-CTGGCTTGTTCAACCTGTCA-3’(Forward), 5’CAACCGTGATCTGTCTGTTAGTC-3’(Reverse); β-Actin:5’-TGTCCACCTTCCAGCAGATGT-3’(Forward), 5’- AGCTCAGTAACAGTCCGCCTAG-3’(Reverse).

### 2.12 Western blotting

To evaluate the changes in protein expression by Western blot, cells were lysed with lysis buffer and the protein concentration was determined by a bicinchoninic acid (BCA) protein assay kit (Sigma, #71285-M). Protein lysates (40μg) were separated by sodium dodecyl sulfate polyacrylamide gel electrophoresis (SDS-PAGE) and electro-transferred to polyvinylidene difluoride (PVDF) membranes. The membrane was blocked in Trisbuffered saline plus tween-20 containing 5% nonfat powdered milk for 1 h and incubated with primary antibodies such as FDX1 (Affinity, #DF7950) and β-actin (Bioss, #bs-0061R) as well as HRP-conjugated secondary antibodies. HRP luminescence was detected with an enhanced chemiluminescence (ECL) detection kit (Meilunbio, #MA0186). The expression of the target band relative to the loading control was quantified with integrated density by ImageJ software.

### 2.13 Fe-S cluster content assay

The levels of Fe-S cluster in cells were determined by double antibody sandwich method using Fe-S Cluster Immunoassay Kit (YJBio, #202312). And the experiments were carried out according to the manufacturer’s instructions. Cells were homogenized with lysis buffer and the Fe-S cluster in samples reacts with reagent to generate adduct which can be quantified colorimetrically.

### 2.14 Statistical analysis

This study utilized the Kaplan-Meier method to determine the overall survival (OS) and log-rank tests to identify significant differences. To compare two groups’ differences, chi-square, corrected chi-square, and Fisher’s exact tests were used for continuous data. Mann-Whitney U was used to compare non-parametric variables. We compared tumor and nontumor tissue gene expression using the Student’s t-test. Through univariate Cox regression analyses, we calculated hazard ratios (HRs) for CRGs by using Wald tests, and then identified independent prognostic factors. We then used the forestplot R package to visualize their independent prognostic value, which was then tested by multivariate analysis. The statistical analyses were performed using R (version 3.5.1) or SPSS Statistics 26.0 (IBM, Armonk, NY, USA). All data from experiment are presented as the mean ± standard error of the mean (SEM). The differences between groups were analyzed using Student’s t-tests or one-way analysis of variance (ANOVA), and *P* < 0.05 was considered statistically significant.

## 3 Results

### 3.1 The prognostic relevance analysis of CRGs in pan-cancer

**Figure 1:**
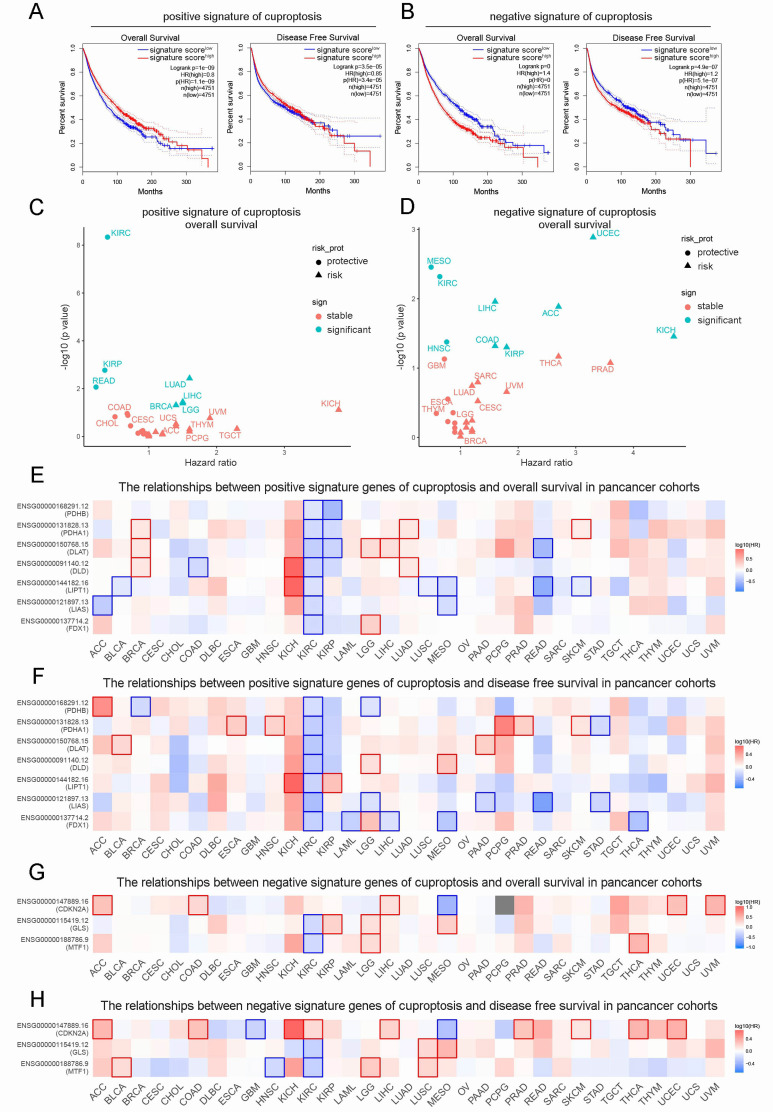
Prognostic appraisal of the CRGs signature in pan-cancer. **(A, B)**. The correlation of pos.cu.sig genes **(A)** and neg.cu.sig genes **(B)** with OS and DFS in pan-cancer. **(C, D)**. The HR and difference significance of pos.cu.sig genes **(C)** and neg.cu.sig genes **(D)** on OS in all TCGA cancer types. **(E, F)**. The correlation of each pos.cu.sig genes (n=7) with OS **(E)** and DFS **(F)** in pan-cancer (Borders represent statistically significant differences). **(G, H)**. The correlation of each neg.cu.sig genes (n=3) with OS **(G)** and DFS **(H)** in pan-cancer (Borders represent statistically significant differences).

We extracted 10 CRGs from published articles, including 7 positive cuproptosis signature(pos.cu.sig) genes (FDX1, DLD, LIPT1, LIAS, PDHA1, DLAT and PDHB) and 3 negative cuproptosis signature(neg.cu.sig) genes (CDKN2A, GLS and MTF1) (1). Among 9502 pan-cancer patients from the TCGA cohort, high expression of pos.cu.sig gene was interrelated with higher survival rates, but neg.cu.sig genes were associated with a poor survival rate (**Figures 1A, B**). Next, we compared OS of pos.cu.sig genes and neg.cu.sig genes in each cancer type, the pos.cu.sig genes of kidney renal clear cell carcinoma (KIRC) were extremely related to prognostic value (**Figures 1C, D**). Then, we further analyzed the relationships between CRGs and OS or disease-free survival (DFS) in pan-cancer cohorts. Results showed that all pos.cu.sig genes were significantly negatively interrelated the HR of OS and DFS in KIRC, however, the consistent change were not found in neg.cu.sig genes (**Figures 1E-H**).

### 3.2 The expression and prognostic characteristics of CRGs in KIRC

**Figure 2:**
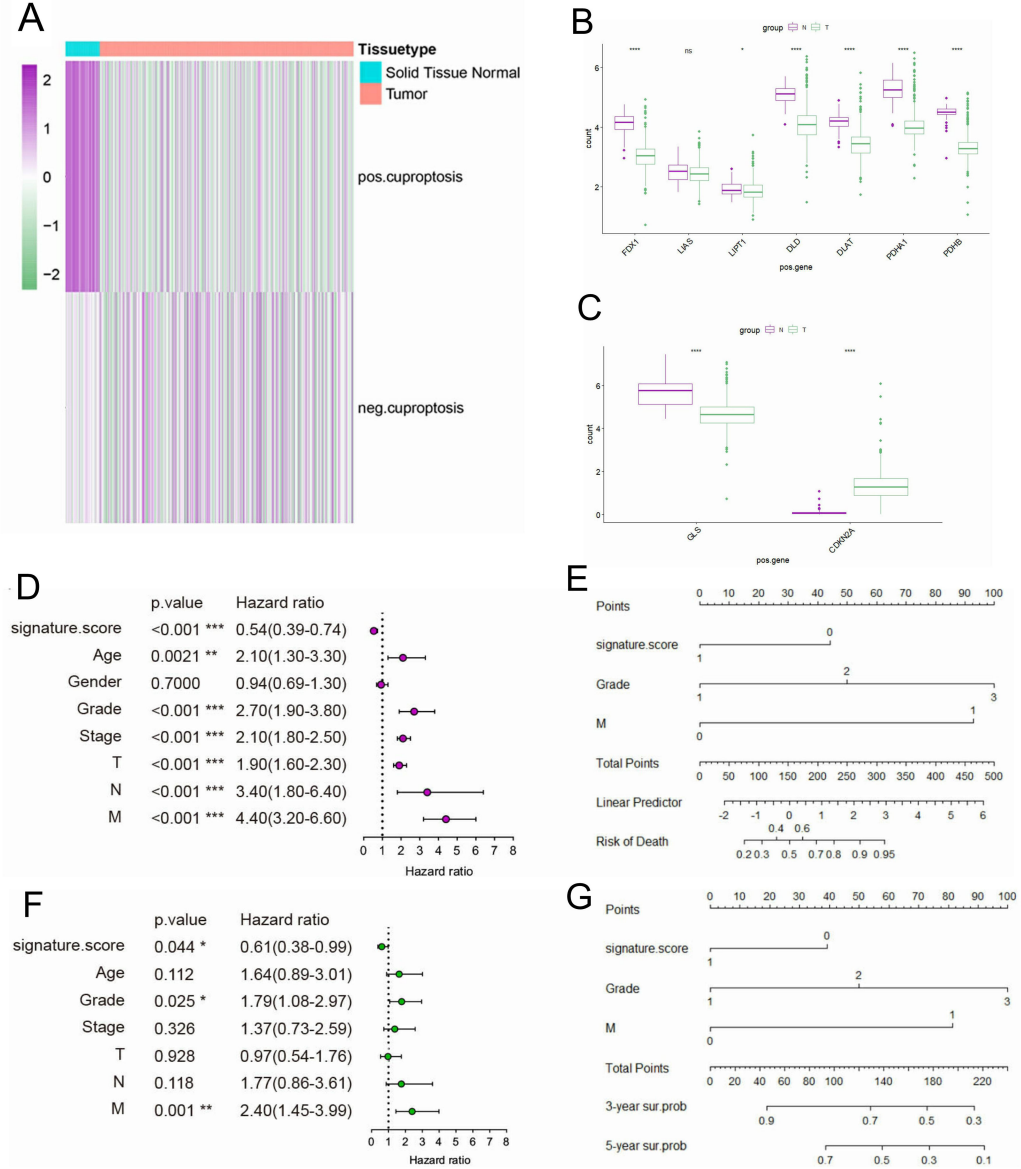
The expression of CRGs in KIRC and its prognostic characteristics. **(A)**. The expression enrichment analysis of CRGs. **(B, C)**. Expression level of 7 pos.cu.sig genes **(B)** and 2 neg.cu.sig genes **(C)** in KIRC and normal tissue. (“ns” means they are not significant; *, *P <*0.05; **, *P* < 0.01; ***, *P* < 0.001; ****, *P <*0.0001). **(D)**. Forest plot of univariate Cox regression analysis. **(E)**. Nomogram for predicting risk of death. **(F)**. Forest plot of multivariate Cox regression analysis. **(G)**. Nomogram for predicting survival probability of 3-and 5-years.

To explore the expression differences of CRGs between normal tissue and KIRC, we proceed Gene Set Variation Analysis (GSVA) enrichment analysis. Interestingly, pos.cu.sig genes were downregulated in KIRC tissues contrast with normal adjacent, while there is no difference in neg.cu.sig genes (**Figure 2A**). At the same time, we showed the expression level of 7 pos.cu.sig genes and 2 neg.cu.sig genes (MTF1 gene expression is lost) in KIRC and normal tissue by box plots. **Figure 2B** showed that FDX1, DLD, LIPT1, DLAT, PDHA1 and PDHB downregulated in KIRC. While 2 neg.cu.sig genes showed discordant changes in KIRC, with GLS down-regulated and CDKN2A up-regulated (**Figure 2C**). Univariate Cox regression analysis indicated that Age, Grade, Stage, and TNM stages were prognostic factors for KIRC. Besides, pos.cu.sig GSVA score were significantly associated with excellent survival (HR=0.54, *P*<0.001) (**Figure 2D**). In addition, the HR of the pos.cu.sig GSVA score determined by multivariate Cox regression analysis was 0.61 (*P* < 0.001) (**Figure 2F**), forecasting that the pos.cu.sig genes were an independent prognostic factor for KIRC. At the meantime, the pos.cu.sig genes had excellent predictive capability for risk of death and 3- and 5- year survival probability (**Figures 2E-G**).

### 3.3 Functional analyses in the TCGA cohort

**Figure 3:**
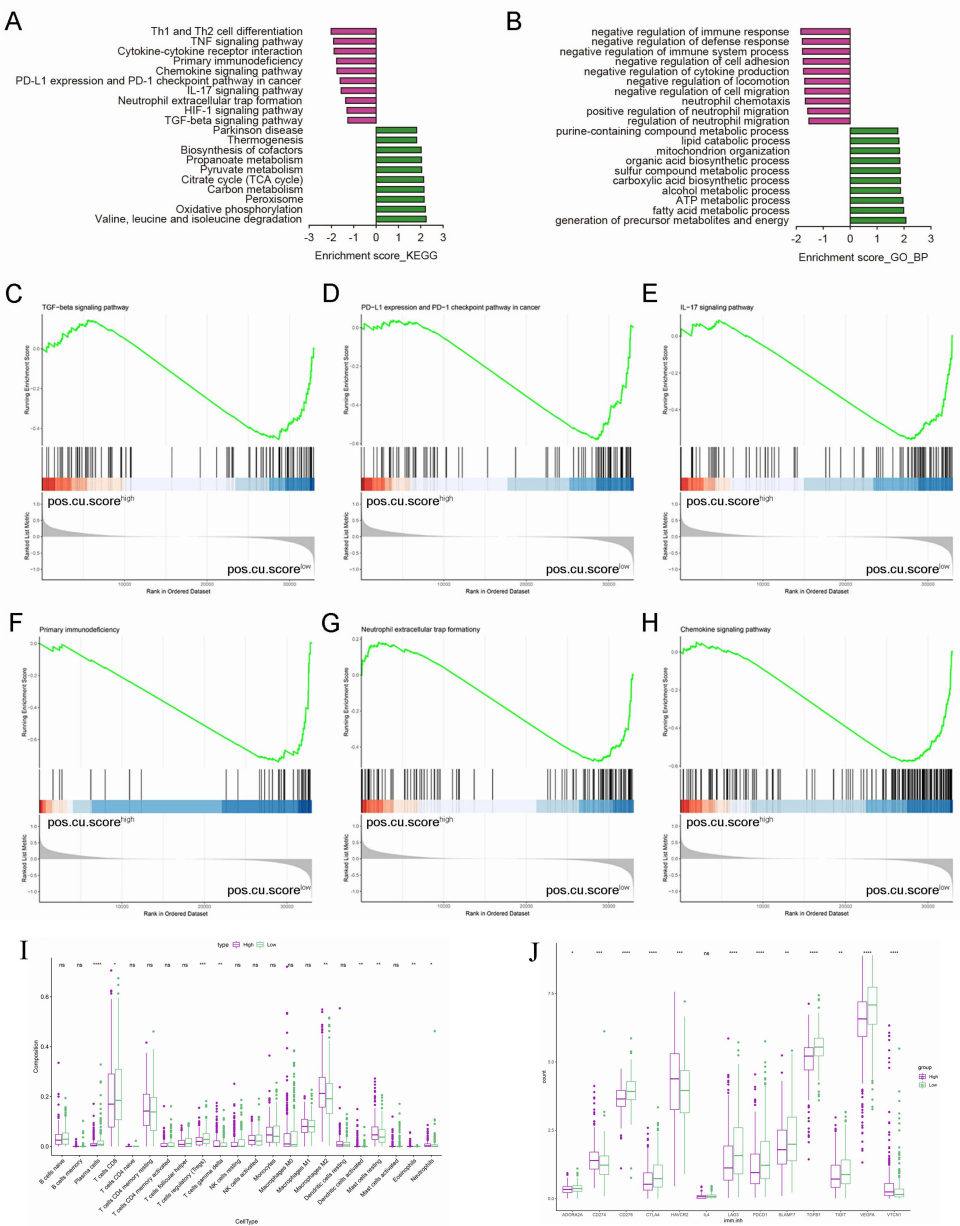
Representative results of KEGG and GO analyses and the results of GSEA. **(A)**. KEGG pathway analyses of the DEGs. **(B)**. GO terms of the DEGs. **(C-H)**. An association of the signature with multifarious signaling pathways is observed. **(I)**. Boxplots show the results of 22 immune-related activities. **(J)**. The scores of 13 immunosuppressive molecules are displayed in boxplots. (“ns” means they are not significant; *, *P* < 0.05; **, *P* < 0.01; ***, *P* < 0.001; ***, *P* < 0.0001).

KEGG pathway and GO enrichment analyses were used to determine which biological functions and pathways are associated with pos.cu.sig scores. DEGs of low-score group were obviously enriched in tumor immunosuppressive microenvironment, such as Th1 and Th2 cell differentiation, TNF signaling pathway, Cytokine-cytokine receptor interaction, PD-L1 expression and programmed death-1 (PD-1) checkpoint pathway in cancer, TGF-beta signaling pathway and negative regulation of immune response (**Figures 3A, B**). Our next step was to use GSEA to elucidate the possible biological mechanisms involved in the progression of KIRC disease. **Figures 3C-H** showed that TGF-beta signaling pathway, PD-L1 expression and PD-1 checkpoint pathway in cancer, IL-17 signaling pathway, Primary immunodeficiency, Neutrophil extracellular trap formationy and Chemokine signaling pathway were activated in the low-score group. Our study further examined the correlation between pos.cu.sig scores and immunity status in KIRC using ssGSEA. Comparing to high-score group, plasma cells, T cells CD8, T cells regulatory (Tregs) in the low-score group were higher (P<0.05, **Figure 3I**). Consistent with the GO enrichment results, various immunosuppressive molecules in the low-score group, including cytokines and inhibitory receptors, were significantly up-regulated in the KIRC tumor microenvironment (*P*<0.05, **Figure 3J**).

### 3.4 FDX1 expression was correlated with prognosis in KIRC

**Figure 4:**
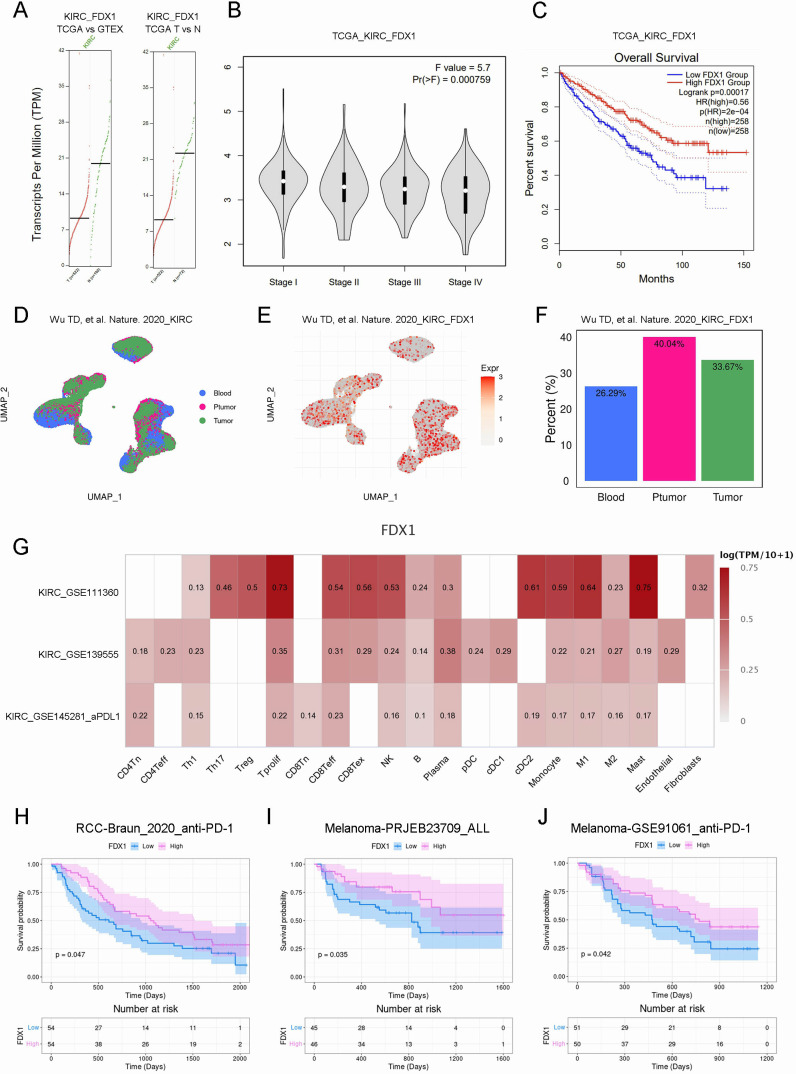
FDX1 expression was correlated with prognosis in KIRC patients. **(A)**. The mRNA expression of FDX1 in normal or cancer tissues. **(B)**. The violin plot showed the correlation. **(C)**. The correlation of FDX1 with OS in KIRC. **(D-F)**. The UMAP analyze of FDX1 expression of single-cell sequencing samples. **(G)**. Heat map showed the correlation. **(H-J)**. The Kaplan–Meier survival curve showed the survival probability.

FDX1 is a key protein in the good control of cuproptosis, to assess the relationship between FDX1 and KIRC prognostic, we investigated FDX1’s expression and prognostic features in KIRC. In this study, we compared FDX1 mRNA levels in TCGA_KIRC and GTEX normal kidney tissues and in TCGA_KIRC and TCGA para-cancerous tissues. In KIRC tissues, FDX1 expression was significantly lower than in normal or para-cancerous tissues (**Figure 4A**). Further, FDX1 expression was significantly correlated with KIRC grade, while low FDX1 expression was related to high pathological grade (P<0.001, **Figure 4B**). Besides, we found a strong correlation between high FDX1 expression and excellent OS through correlation analysis (P<0.001, **Figure 4C**). Accordingly, FDX1 expression was positively associated with prognosis in KIRC patients. (**Figures 4D-F**). What is noteworthy is that the level of FDX1 mRNA increased in KIRC tissue after anti-PD-L1 treatment, indicating that a higher FDX1 level results in a more effective immunotherapy (**Figure 4G**). Survival analysis in the literature showed that the higher the level of FDX1 mRNA, the better the anti-PD-1 treatment effect in not only KIRC but also melanoma (7) (**Figures 4H-J**).

### 3.5 FDX1 may be involved in cuproptosis of Caki-1 cells induced by Elesclomol

**Figure 5:**
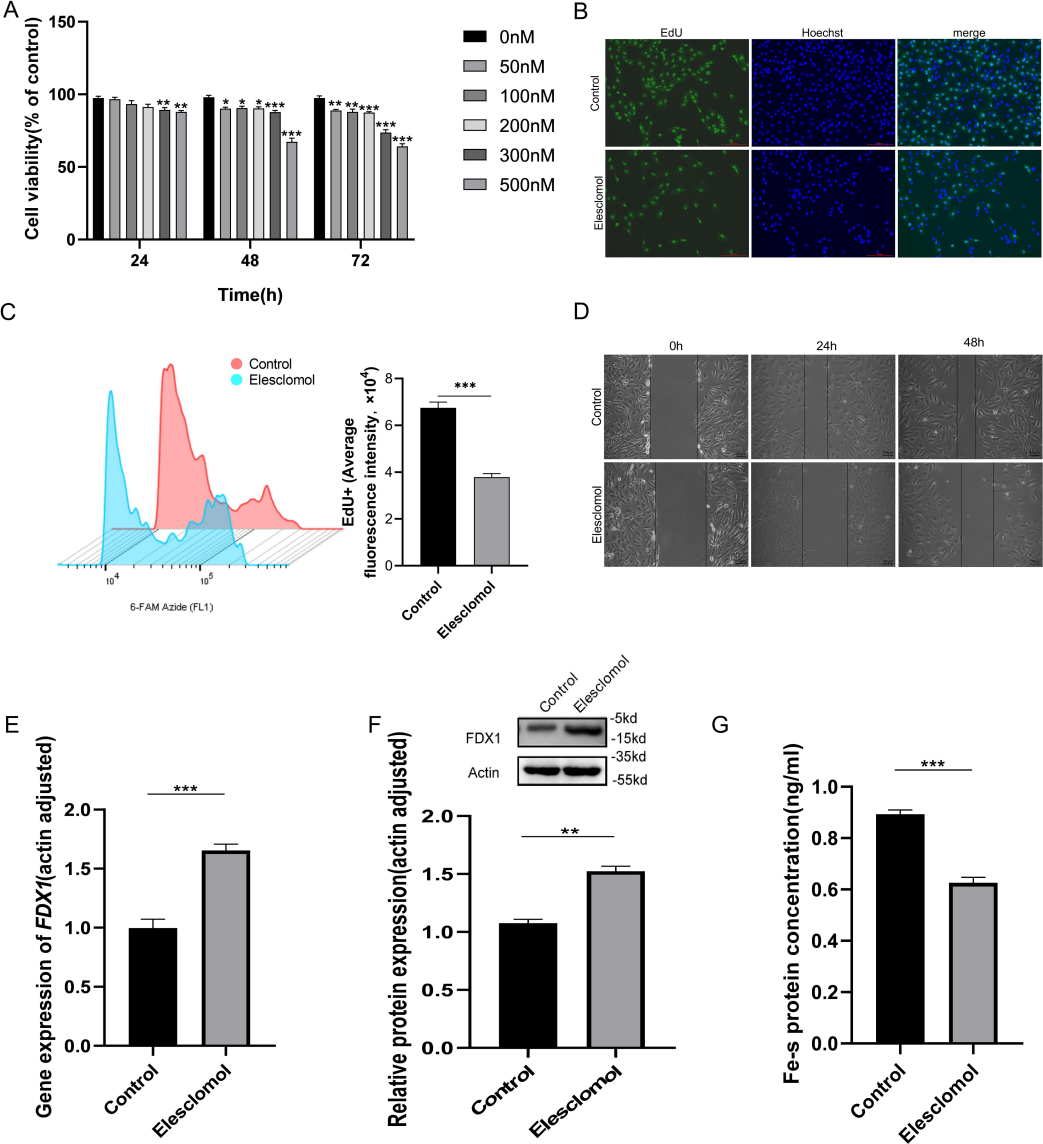
Effect of Elesclomol on proliferation and migration of KIRC cells and regulation of FDX1. **(A)**. Caki-1 cells viability was measured using the CCK-8 kit after treating with Elesclomol for 24, 48,72h. **(B)**. EdU staining of Caki-1 cells. Scale bar, 200 μm; n=3; **(C)**. Caki-1 cell EdU flourescence intensity analysis by flow cytometry. **(D)**. The healing/closing state of Caki-1cells by the scratch assay (representative pictures from 3 repeated experiments). **(E, F)**. *FDX1* mRNA **(E)** and protein **(F)** expression level of Caki-1 cells treated with Elesclomol (500nM) for 48h. **(G)**. The content of Fe-S cluster was tested of Caki-1 cells c treated with Elesclomol (500nM) for 48h. Experiments were repeated three times and the data are expressed as the mean ± SEM. *P < 0.05, ** *P <*0.01 and *** *P* < 0.001.

To investigate the effect of Elesclomol on KIRC cells (Caki-1 cells) proliferation and whether FDX1 is involved in regulation. We firstly access the effect of different concentrations Elesclomol (50, 100, 200, 300, 500nM) on Caki-1 cells viability. CCK-8 results showed that the cell viability of Caki-1 cells was decreased after Elesclomol treat for 48h and 72h, and higher concentrations were progressively less viability, but not appear in 24h (**Figure 5A**). Compared with control group, the EdU staining (**Figure 5B**) and EdU+ average fluorescence intensity (**Figure 5C**) of Caki-1 cells significantly decreased after Elesclomol (500nM) treating for 48h. The scratch assay was used to study the effects of Elesclomol on the migration of Caki-1 cells. The control cells gradually healed after incubating for 24 and 48 hours, while Elesclomol treated cells showed a lower “wound” heal speed after incubation for 24 and 48 hours (**Figure 5D**). Then qRT-PCR and WB were performed to detect the expression changes of FDX1 mRNA and protein in Caki-1 cells after Elesclomol (500nM) treating for 48h. The results showed that the *FDX1* gene expression in Caki-1 cells were remarkably elevated after Elesclomol treating (**Figures 5E, F**, *P*<0.05). FDX1 can mediate Fe-S cluster biosynthesis, Fe-S cluster protein reduction is one of the characteristics of cuproptosis (8). Therefore, we sought to further evaluate the Fe-S cluster protein content of Caki-1 cells after Elesclomol treating. The Fe-S cluster protein content of Elesclomol treated Caki-1 cells decreased significantly compared with the control group (**Figure 5G**).

## 4 Discussion

Cuproptosis (1), a new cell death method named in 2022, which is obviously different from the known regulated cell death methods such as apoptosis, necroptosis, pyroptosis and ferroptosis. According to research, cuproptosis is closely associated with cancer development and prognosis. Our research focused on the correlation between CRGs and of pan-cancer patients based on data extracted from the TCGA database. The OS and DFS of pan-cancer patients was better in pos.cu.sig high-score group, but not in pos.cu.sig low-score group, which indicated that pos.cu.sig maybe a protective factor in tumor patients and plays a tumor suppressor role, while neg.cu.sig generally promotes tumor growth, which was consistent with a recent study that reported that the cuproptosis level may influence the survival of patients with different tumor types (9).

To determine the correlation of CRGs and the prognosis of specific cancers, we analyzed the association of pos.cu.sig and neg.cu.sig with OS in various cancer types. To our surprise, pos.cu.sig genes can not only be used as a protective factor for KIRC, but also has the most significant prognostic significance in KIRC. In this study, each gene of pos.cu.sig was significantly negatively correlated with HR of OS and DFS in KIRC. Besides, all the pos.cu.sig genes were differentially expressed between KIRC and adjacent nontumorous tissues. These results prompted us to focus on the potential role of cuproptosis in KIRC and the possibility of building a prognostic model with these CRGs. Our hypothesis that CRGs may be involved in the regulation of the antitumor process is supported by the findings of the univariate Cox regression analysis, which showed that pos.cu.sig genes were significantly related to the survival time of KIRC patients. Following that, multivariate Cox regression demonstrated that a new prognostic model incorporating 7 pos.cu.sig genes can separate patients with poor prognosis from those with good prognosis. Compared to the widely used clinical stage, grade, and M stage, this prognostic model performed better for KIRC.

KIRC is one of the most common malignancies, advanced KIRC has an extremely poor prognosis due to its inherent resistance to radiotherapy and chemotherapy (10). KIRC is a highly immune-infiltrating tumor and one of the earliest malignancies to respond to immunotherapy (11). Previous studies have shown that immune cells infiltrating tumor tissue are highly correlated with tumor progression, so they have become the focus of cancer research in recent years (12).Our KEGG and GO analysis results showed that the Th1 and Th2 cell differentiation, TNF signaling pathway, Cytokine-cytokine receptor interaction, PD-L1 expression and PD-1 checkpoint pathway in cancer, TGF-beta signaling pathway were enriched in the low-score group, which suggest that cuproptosis may be closely related to TIME. Studies have shown that high Th1 cell (13) or low TNF (14) recruiting score is related to the poor prognosis of KIRC patients. Previously, PD-L1 has been reported to act as an accurate biomarker for KIRC, and the agents targeting PD-L1 have had promising effects in renal cell carcinoma (15, 16). In this study, the negative regulation of immune response was obviously activated in the low-score group. One speculation is that cuproptotic cells may regulate the progress of the tricarboxylic acid cycle (TCA) to affect the activity of immune cells (1, 17). Besides, the increases of T regulatory cells (Tregs), and the decreases of T cells gamma delta (γδ T cells) and dendritic cells activated represent immunosuppressive status in low-score group patients. It is reported that a large number of Treg cells exist in the tumor microenvironment, Treg cells can inhibit the function of effector T cells and play a key role in tumor immune escape (18). The prognosis of patients with γδ T cell infiltrating tumor is better than that of patients with other immune cell infiltrating tumors (19). γδ T can not only kill tumor cells directly, but also activate B/DC/αβ T/NK cells indirectly kill tumor cells, so γδ T cells have become a new research focus in cellular immunotherapy (20). Activated DC cells can trigger the specific immune responses and play an anti-tumor role. Therefore, the weakened antitumor immunity of low-score group patients may lead to KIRC patient poor prognosis. Whether these CRGs play a role in KIRC patients’ prognosis by influencing the process of tumor immunity remains to be elucidated, the specific mechanism needs further study.

FDX1 is a ferredoxin in the human mitochondria, studies have confirmed that FDX1 is a key factor for cuproptosis by regulating protein fatty acylation (21). Deletion of FDX1 resulted in the functional loss of protein fatty acylation, the accumulation of pyruvate and α-ketoglutarate and the depletion of succinate in cells, indicating that the functional loss of protein fatty acylation blocks the progression of the TCA (22). Since our previous results have shown that the downregulation of pos.cu.sig is closely related to the formation of the tumor immunosuppressive microenvironment in KIRC, we were very curious about the prognostic characteristics of the expression of FDX1 as a cuproptosis core gene in KIRC, and whether it is related to tumor immunotherapy. Consistent with the literature (23), the results showed that FDX1 was underexpressed in KIRC and associated with poor prognosis. More importantly, the immunotherapy data suggested that FDX1 can serve as a potential predictor for the assessment of immunotherapy efficacy in KIRC, with higher FDX1 expression levels leading to better immunotherapy efficacy, which was confirmed in two other melanoma cohorts (7, 24). Then, we conducted cell experiments to verify whether KIRC cells can be induced to cuproptosis by Elesclomol and whether FDX1 is involved in the regulation. Elesclomol is a potent copper ionic carrier that promotes cuproptosis (25). The results of our cell experiments show that Elesclomol can obviously suppressed the cell proliferation and migration of Caki-1 cells. In addition, Elesclomol specifically binds to the α2/α3 helix and β5 chain of FDX1 and inhibits FDX1-mediated Fe-S cluster biosynthesis. Interestingly, the level of mRNA and protein of *FDX1* was up-regulated and Fe-S cluster protein was decreased in Caki-1 cells after Elesclomol treatment. We further inferred that the *FDX1* up-regulation is negatively feedback by the content decrease of Fe-S. In conclusion, the key regulatory gene FDX1 of pos.cu.sig genes can be used as a candidate marker and potential therapeutic targets for tumor immunotherapy prognosis evaluation in KIRC.

## 5 Conclusion

As a result, we developed a new prognostic model using 7 CRGs that has been shown to be independently related to OS and can precisely forecast the prognosis of KIRC. Determining the therapeutic targets for KIRC can be aided by understanding the underlying mechanisms and importance of these CRGs in KIRC. Further research is necessary to better understand the underlying mechanisms relating CRGs to tumor immunity in KIRC.

## Data Availability

All data generated or analyzed during the present study were downloaded from TCGA database (https://www.cancer.gov/ccg/research/genome-sequencing/tcga). The RNA-Seq datasets used in this study is based on the UCSC Xena project (https://xenabrowser.net/datapages/), which are computed by a standard pipeline.
